# Iron and Virulence in *Stenotrophomonas Maltophilia*: All We Know So Far

**DOI:** 10.3389/fcimb.2018.00401

**Published:** 2018-11-12

**Authors:** V. Kalidasan, Narcisse Joseph, Suresh Kumar, Rukman Awang Hamat, Vasantha Kumari Neela

**Affiliations:** Department of Medical Microbiology and Parasitology, Faculty of Medicine and Health Sciences, Universiti Putra Malaysia, Selangor Darul Ehsan, Malaysia

**Keywords:** *S. maltophilia*, iron-depleted, Fur, siderophore, microbial iron acquisition, virulence factors

## Abstract

*Stenotrophomonas maltophilia* is a multi-drug-resistant global opportunistic nosocomial pathogen, which possesses a huge number of virulence factors and antibiotics resistance characteristics. Iron has a crucial contribution toward growth and development, cell growth and proliferation, and pathogenicity. The bacterium found to acquire iron for its cellular process through the expression of two iron acquisition systems. Two distinct pathways for iron acquisition are encoded by the *S. maltophilia* genome-a siderophore-and heme-mediated iron uptake system. The *entAFDBEC* operon directs the production of the enterobactin siderophore of catecholate in nature, while heme uptake relies on *hgbBC* and potentially *hmuRSTUV* operon. Fur and sigma factors are regulators of *S. maltophilia* under iron-limited condition. Iron potentially act as a signal which plays an important role in biofilm formation, extracellular polymeric substances (EPS), extracellular enzymes production, oxidative stress response, diffusible signal factor (DSF) and siderophore production in *S. maltophilia*. This review summarizes the current knowledge of iron acquisition in *S. maltophilia* and the critical role of iron in relation to its pathogenicity.

## Introduction

*Stenotrophomonas maltophilia* is a Gram-negative, Gammaproteobacteria, that is present ubiquitously in the environment; particularly in the soil and plants rhizospheres (Alavi et al., 2014). Therefore, *S. maltophilia* has many attributes that could be applied in different biotechnological processes such as bioremediation, phytoremediation, degradation of an organic compound, biocontrol activity and many more (Antonioli et al., 2007; Pages et al., 2008; Mukherjee and Roy, 2016). Despite its biotechnological applications, the bacterium was recently reviewed to gain access into the clinical settings, thus recognized as an important multi-drug-resistant global opportunistic nosocomial pathogen (Brooke et al., 2017). *Stenotrophomonas maltophilia* is responsible for causing various infections ranging from bacteremia, endocarditis, pneumonia, meningitis, ocular infections, urinary tract infection, enteritis, and skin/soft tissue infections (Senol, 2004; Abbott et al., 2011). A debatable question regarding “*S. maltophilia* is a colonizer or a pathogenic culprit?” still remains due to the failure in distinguishing colonization and acquired infections, as the microorganism poses a limited pathogenic potential in causing illness in healthy hosts (Neela, 2014; Norton and Dachs, 2015).

The invading pathogen must be able to produce various virulence factors in order to establish infections and this largely depends on environmental conditions and level of micronutrients within the hostile environment (Sritharan, 2006). In such circumstances, *S. maltophilia* is known to exhibit its pathogenicity through: (1) pili/flagella/fimbrial/adhesins which contributes to adherence, auto-aggregation, colonization of biotic and abiotic surfaces; (2) outer membrane lipopolysaccharide (LPS) plays a role in biofilm formation and resistance to antibiotic as well as complement-mediated cell killing; (3) diffusible signal factor (DSF) plays a huge role in quorum sensing, which in turn mediate motility, extracellular enzymes production, LPS synthesis, microcolony formation, and tolerance toward antibiotics and heavy metal ions; and (4) extracellular enzymes production such as proteases, lipases, esterase, DNase, RNase, and fibrinolysin (Looney, 2005; Abbott et al., 2011; Brooke, 2012).

In general, most of the bacteria can acquire all of the nutrients such as nitrogen, amino acids, nucleotides, phosphates and other inorganic ions for its survival, except for iron as it is not freely available from the host tissue (Ratledge and Dover, 2000). In order to counteract the difficulty to fulfill the iron requirement, the bacteria have evolved numerous mechanisms; particularly by demonstrating efficient iron acquisition systems under iron-limited conditions (Andrews et al., 2003; Thomas and Wigneshweraraj, 2014; Kalidasan et al., 2018b). This phenomenon is not an exception for *S. maltophilia*, as iron was found to plays a crucial role in the regulation of its virulence activities (García et al., 2015). At this juncture, we highlight the iron acquisition strategies in *S. maltophilia* focusing on the siderophore- and heme-mediated systems; together describing the regulator involved in iron homeostasis and metabolism. The expression of virulence factors in relation to iron availability in *S. maltophilia*, is discussed extensively in this review.

## Iron acquisition systems in *S. maltophilia*:

**Figure 1:**
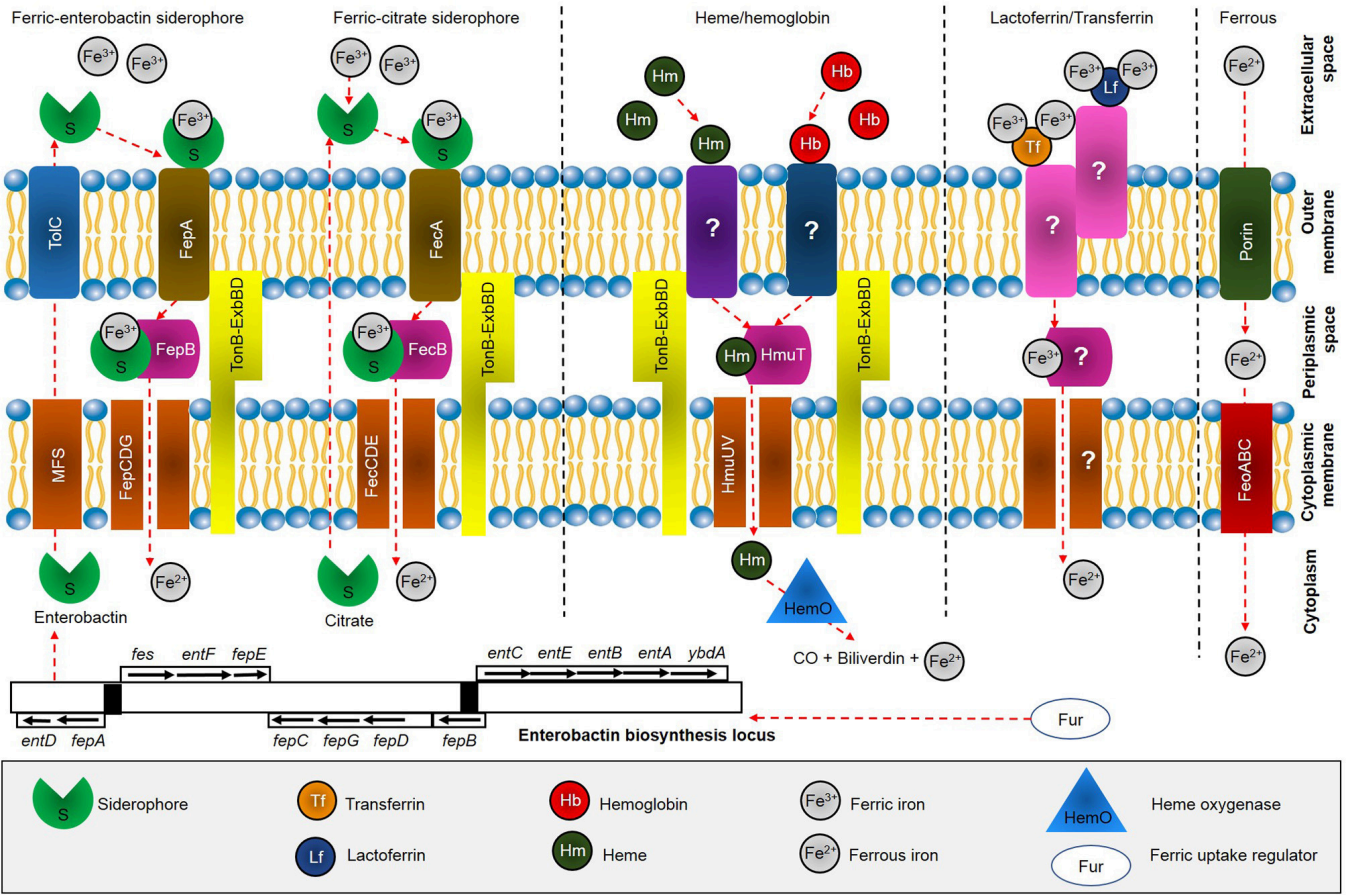
Overview of iron acquisition systems in *S. maltophilia*. After biosynthesis, siderophore enterobactin is effluxes from cytoplasm through major facilitator superfamily (MFS) protein and further into the extracellular space by outer membrane factor TolC. Enterobactin scavenges free Fe^3+^ available at the extracellular space and is subsequently recognized and taken up through FepA, which is energized by the TonB-ExbBD machinery. FepB delivers ferric enterobactin from the periplasm by FepCDG transporter into the cytoplasm. On the other hand, ferric citrate is recognized by FecA and further delivered into periplasmic by FecB and transported across the cytoplasm by FecCDE transporter. Heme acquisition is predicted to be taken through receptor at the outer membrane, followed by HmuTUV system. Uptake of iron bound to transferrin and lactoferrin have not been fully identified (marked ?), while Feo system involved in uptake of ferrous iron through action of FeoABC.

Little is known about iron uptake systems in *S. maltophilia* (Huang and Lee Wong, 2007). However, the iron acquisition strategies in other Gram-negative bacteria have been extensively studied previously (Braun and Hantke, 2013; Runyen-Janecky, 2013). In general, the iron uptake systems in Gram-negative bacteria can be mediated by: (1) transferrin (Tf) or lactoferrin (Lf); (2) heme (Hm) and hemoglobin (Hb); (3) siderophores; and (4) ferrous iron (Fe^2+^) (Marx, 2002). The bacteria depends on high-affinity surface receptor proteins that potentially bind with ferric iron (Fe^3+^) loaded to siderophores or heme, and followed by subsequent delivery into the periplasmic space by the TonB–ExbB-ExbD complex (Faraldo-Gómez and Sansom, 2003). The periplasmic-binding proteins and ATP transporters available at the cytoplasmic membrane are used to ensure further transport into the cell. On the other hand, Hm can be obtained from Hb and hemoglobin-haptoglobin (Hb-Hpt) complex by outer membrane proteins (OMPs). Apart from that, some Gram-negative bacteria can utilize Fe^3+^ bound to transferrin and lactoferrin at the outer membrane, and transported into the cell. Under anaerobic conditions, soluble Fe^2+^ can diffuse across outer membrane porins, and is subsequently imported by FeoABC system. A model for iron uptake in *S. maltophilia* can reasonably be proposed based on previous studies (Adamek et al., 2014; Nas and Cianciotto, 2017; Kalidasan et al., 2018a) as shown in Figure 1.

Although *S. maltophilia* was previously reported to uptake iron through pseudobactin (Jurkevitch et al., 1992), a siderophore produced by *Pseudomonas* strain B10 (Teintze et al., 1981), it was not clear whether the bacterium is capable of producing its own siderophores (Kumar and Audipudi, 2015). Furthermore, the gene(s) responsible for iron acquisition through siderophores is still a question (Adamek et al., 2014). In the study, *S. maltophilia* isolates K279a and SKK35 (clinical strains), R551-3 (environmental strain), SKA14 (seawater strain), and RA8 (wastewater strain) were found to harbor genes *entACF* encoding for enterobactin synthetase, that catalyzes the biosynthesis of enterobactin siderophore. However, the siderophore production that can only function in combination with other genes should be interpreted in the context of presence of those other genes; i.e., incomplete gene sets (*entBDE*) for biosynthesis of enterobactin in *S. maltophilia*. A recent study revealed the presence of eight loci in *S. maltophilia* K279a, which are predicted to encode a system for siderophore production, as shown in Figure 1 (Nas and Cianciotto, 2017). The first locus had six open-reading-frames (ORFs) needed to make enterobactin including, EntA, EntF, EntD, EntB, EntE, and EntC, with addition of major superfamily (MFS) membrane transport protein. The second locus encodes TolC which mediates siderophore export across the outer membrane, while the third locus encodes enterobactin receptor FepA. The periplasmic-spanning complex TonB, ExbB, and ExbD proteins were encoded by locus four, five and six, respectively. The seventh locus encodes proteins with similarity to FepC, FepD, and FepG, while the last locus encodes YgiH and ViuB which assist the release of iron from other siderophores. The study concluded *S. maltophilia* produces an EntC-dependent catecholate siderophore that is distinct from enterobactin, as the siderophore appeared to have a modification at position-3 and/or position-4 in the catecholate structure. The claim was achieved through numerous investigations, such as inability of K279a supernatants to restore growth of *Salmonella typhimurium* enterobactin-indicator strain (TA2700) on a low-iron medium; ability of K279a siderophore extraction into ethyl acetate but not butanol and dichloromethane; inability of K279a siderophore to migrate as far as enterobactin in thin-layer chromatography (TLC) indicating it is more polar than enterobactin; and a mixture of enterobactin and its monomer did not stimulate the growth of K279a or its *entC* mutant and *fepA* mutant derivatives.

Furthermore, mass spectrometry analysis in *S. maltophilia* K279a identified SMLT_RS06850 and SMLT_RS19685 encoding for outer membrane receptor FepA and TonB-dependent receptor respectively (García et al., 2015). A BLAST identity revealed SMLT_RS06850 displays similarity of 66% to *Xanthomonas citri*, while SMLT_RS19685 was found 55% similarity with *Pseudomonas putida*. In short, genomic investigations suggested *S. maltophilia* potentially secrete catecholate siderophore and depending on *entABCDEF* operon for production of distinct enterobactin. On the other hand, plant-associated strains *S. maltophilia* R551-3 and *Stenotrophomonas rhizophila* DSM14405 were found to harbor iron uptake locus *fcuA* and *fhuA* encoding for ferrichrome receptor proteins, which code for siderophore receptors and the outer membrane adhesin-like gene, respectively (Alavi et al., 2014). It is worthy to noted that, the structure and mechanisms of the outer membrane transporter of enterobactin (*fepA*), is closely similar to that of FhuA (Marx, 2002).

Siderophores are small molecules and considered to be an important virulence factor, particularly in pathogens that encode multiple siderophores (Holden and Bachman, 2015; Behnsen and Raffatellu, 2016). Any pathogenic strains that are capable of over-producing siderophores are considered to be hypervirulent, whereas strains unable to secrete siderophores have decreased virulence and fitness during infection and colonization. As a far concern, siderophore production in *S. maltophilia* has been well studied in recent years. Siderophore production among *S. maltophilia* in the rhizosphere of oilseed rape, showed all isolates investigated were positive for siderophore activity, ranging from 5 to 20 mm orange zone on CAS agar (Berg et al., 1996). In contrast, *S. maltophilia* strain W81 did not produce prominent fluorescent siderophores (Dunne et al., 1997). The variation in siderophore production, particularly among environmental isolates were also observed in our study (Kalidasan et al., 2018a). We noted the environmental strains did not produce siderophores or produced very minimal amounts compared to clinical isolates investigated. We also observe the percentage of siderophore production investigated through liquid CAS, showed clinical isolate produced a greater amount of siderophore (30.8%) compared to environmental isolate (4%).

Furthermore, an analysis of 50 isolates comprised of clinical and environmental strains was reported to produce minimum siderophore activity, ranging from 5 to 3 mm orange zone on CAS agar (Minkwitz and Berg, 2001). On the hand, analysis of all 32 clinical isolates of *S. maltophilia* showed siderophore activity ranging from 4.5 to 11 mm orange zone on modified CAS agar and secretion of catechol-type siderophores (Garcia et al., 2012). Similarly, both clinical and environmental isolates produced catechol-type enterobactin (Ryan et al., 2009), also supported by cross-feeding assay in *S. maltophilia* (Mokracka et al., 2011). Aforementioned, *S. maltophilia* secretes catecholate siderophore that appears to be novel in structure, rather than enterobactin (or salmochelin) (Nas and Cianciotto, 2017). Although most of the studies reported *S. maltphilia* is a catecholate-type siderophore producer, a contrary investigation showed *S. maltophilia* clinical isolates were a hydroxamate-type ornibactin producer, as the study lack estimation of catecholate derivatives (Chhibber et al., 2008). Ornibactin was reported being produced by *Burkholderia cepacia* complex (BCC) (Sokol et al., 2000; Visser et al., 2004), and such production is possible as *S. maltophilia* and BCC are a closely related group of non-fermenting gram-negative bacilli (NFGNBs) (Gautam et al., 2009). However, further investigations are required to confirm whether *S. maltophilia* potentially secretes hydroxamate siderophores under iron limitation.

Even though hemoproteins serve as an iron source for many pathogenic bacteria, heme-acquisition among *S. maltophilia* has not been fully understood yet. *S. maltophilia* isolates were found to harbor gene *hgbBC* encoding hemoglobin binding protein, which suggests potential heme and hemoglobin uptake capability as iron sources (Adamek et al., 2014). However, our previous genotypic and phenotypic investigation identified numerous heme-mediated acquisition system in *S. maltophilia* including: (1) heme oxygenase, associated with heme uptake (HemO/HO); (2) heme ABC transporter, ATPase component (HmuV); (3) hypothetical protein related to heme utilization (Hyp1); (4) heme ABC transporter, permease protein (HmuU); (5) heme ABC transporter, cell surface heme and hemoprotein receptor (HmuT); (6) hemin uptake protein (Hup); and (7) hemin transport protein (Htp) (Kalidasan et al., 2018a). Furthermore, the growth of clinical (SM77) and environmental (LMG10879) isolates was stimulated with Hb and Tf supplementation, while hemin and Lf having less effect in enhancing the growth of the tested isolates. These findings merit further investigations, to decipher how *S. maltophilia* could potentially uptake heme and hemin as iron sources, especially when it is associated with bloodstream infection in human host.

## Regulator of iron acquisition in *S. maltophilia*

In most Gram-negative bacteria, iron homeostasis, metabolism, and virulence is regulated by the ferric uptake regulator protein (Fur), which potentially represses transcription upon interaction with Fe^2+^ or causes de-repression in the absence of Fe^2+^ (Andrews et al., 2003; Troxell and Hassan, 2013). Till date, only study by García et al. (2015) was the first to provide data about the role of iron as a signal, likely through the Fur system in *S. maltophilia*. The study identified 20 putative Fur boxes using MAST tool. However, it is important to note that, there is no evidence of Fur direct regulation, as the study did not demonstrate the binding of the regulator to the promoters of its putative target genes, either by electrophoretic mobility shift assay (EMSA) or DNase footprinting assay. Moreover, our study has only identified the presence of Fur in clinical and environmental isolates of *S. maltophilia* through PCR and significant upregulation of Fur under iron-depleted than under iron-replete conditions, suggesting de-repression of Fur (Kalidasan et al., 2018a). In support of these, regulation of iron uptake system in *S. maltophilia* through Fur was reported in RegPrecise 4.0 database (http://regprecise.lbl.gov/) (Novichkov et al., 2013). The database predicted 17 operons and 39 genes influenced by iron that cater to the pathway for iron homeostasis in *S. maltophilia* strain K279a as shown in Table 1. It is important to mention that, RegPrecise was constructed and manually curated by utilizing the comparative genomic approach, suggesting further analysis and validation. In spite of, our bioinformatics validation revealed, the regulon showed similarities with *P. aeruginosa* strain E15_London_28_01_14, which suggests *S. maltophilia* is closely related to the *Pseudomonas* species (Calza et al., 2003).

**Table 1:** Comparison of regulon of Fur in *S. maltophilia* strain K279a with *P. aeruginosa* strain E15_London_28_01_14.

**Regulon of Fur in** ***S. maltophilia*** **K279a**^**a**^			**Homology (BLAST)**^**b**^	
**Gene**	**Locus tag**	**(Putative) Product**	**Product**	**Identity (%)**
fpvA	SMLT_RS05990	TonB-dependent siderophore receptor	Ferripyoverdine receptor precursor	96
fecI4	SMLT_RS13960	RNA polymerase sigma factor	RNA polymerase sigma factor	40
fecR4	SMLT_RS13965	Iron dicitrate transporter FecR	FecR family protein	32
fecA4	SMLT_RS13970	TonB-dependent receptor	TonB-dependent receptor	48
	SMLT_RS13975	Iron regulated lipoprotein	Hypothetical protein	40
	SMLT_RS13980	Energy transducer TonB	Hypothetical protein	94
fecI	SMLT_RS13545	RNA polymerase sigma factor FecI	Sigma-70 family RNA polymerase sigma factor	53
fecR	SMLT_RS13550	FecR family iron uptake regulator protein	FecR family protein	36
fecA	SMLT_RS13555	Heme-binding protein	Hemin receptor precursor	42
fpr	SMLT_RS15360	Ferredoxin–NADP reductase	Ferredoxin–NADP reductase	98
feoA	SMLT_RS10625	Ferrous iron transport protein A	FeoA domain protein	98
feoB	SMLT_RS10630	Ferrous iron transporter B	Ferrous iron transport protein B	98
	SMLT_RS10635	Hypothetical protein	Hypothetical protein	95
fhuE	SMLT_RS19060	TonB-dependent siderophore receptor	Outer-membrane receptor for Fe(III)-coprogen, Fe(III)-ferrioxamine B and Fe(III)-rhodotrulic acid	87
	SMLT_RS05550	Hypothetical protein	Hypothetical protein	92
bfrA	SMLT_RS05545	TonB-dependent receptor	Colicin I receptor precursor	95
fecI2	SMLT_RS12710	RNA polymerase sigma factor	RNA polymerase sigma factor	53
fecR2	SMLT_RS12715	Transcriptional regulator	FecR family protein	44
fecA2	SMLT_RS12720	TonB-dependent receptor	TonB-dependent receptor	32
hemP	SMLT_RS03780	Hemin uptake protein	Hemin uptake protein	98
hemR	SMLT_RS03785	TonB-dependent hemoglobin/ transferrin/lactoferrin family receptor	Hemin receptor precursor	78
	SMLT_RS03790	Hypothetical protein	Hypothetical protein	93
bfd	SMLT_RS20460	Bacterioferritin	Bacterioferritin-associated ferredoxin	100
bfr	SMLT_RS20455	Bacterioferritin	Bacterioferritin	99
pepSY	SMLT_RS05540	Membrane protein	Putative periplasmic protein	97
	SMLT_RS05535	Hypothetical protein	Putative periplasmic protein	98
	SMLT_RS05570	Hypothetical protein	No significant similarity found
fhuA	SMLT_RS05565	TonB-dependent receptor	Virulence-associated outer membrane protein Vir-90	94
	SMLT_RS05560	sel1 repeat family protein	Polar organelle development protein	97
piuC	SMLT_RS05555	PKHD-type hydroxylase	PKHD-type hydroxylase	98
fecI3	SMLT_RS18585	RNA polymerase sigma factor	Putative RNA polymerase sigma factor FecI	98
fecR3	SMLT_RS18580	Iron dicitrate transport regulator FecR	fec operon regulator FecR	88
fecA3	SMLT_RS18575	Heme-binding protein	Hemin receptor precursor	94
	SMLT_RS13580	TonB-dependent siderophore receptor	Iron(III) dicitrate transport protein FecA	96
pepSY	SMLT_RS07530	PepSY domain-containing protein	Putative iron-regulated membrane protein	95
	SMLT_RS07525	DUF3325 domain-containing protein	Hypothetical protein	87
fhuA	SMLT_RS14400	TonB-dependent siderophore receptor	Virulence-associated outer membrane protein Vir-90	91
pfeA	SMLT_RS06850	TonB-dependent siderophore receptor	Ferric enterobactin receptor precursor	97

a Regulon and locus tag modified from RegPrecise 4.0 (http://regprecise.lbl.gov)

b *All the homologs and identity are corresponding to P. aeruginosa strain E15_London_28_01_14, except those marked in red were obtained from other P. aeruginosa strains*.

Under anaerobic conditions or at low pH, Fe^2+^ is more abundant and in most bacteria, Feo system is dedicated to transport such iron source into the cell (Lau et al., 2015). The Feo system comprised of mainly of FeoA and FeoB proteins, in which FeoA directs to the inner leaflef of the cytoplasmic membrane, where it could possibly interact with FeoB. In *S. maltophilia*, the structure of FeoA adopted Src Homology 3 domain (SH3 domain) fold, containing five antiparallel β-strands, additional α-helices at the N-terminal site, RT loop, and C-terminal β-strand (Su et al., 2010). This novel FeoA forms a unique dimer cross-linked by two zinc ions, which was coordinated by His21 in the RT loop of a molecule and Glu52 in the n-Src loop of another molecule. The center of the RT loop was predicted to be favorable for interacting with metal ions. The study also proposed that FeoA may interact with FeoB between the SH3b domain and G-protein domain in order to regulate FeoB-dependent ferrous iron uptake activity as an activating factor. This SH3 domain have been predicted to act as the targeting domains involved in bacterial cell wall recognition and binding as well as involved in metal-binding (Kamitori and Yoshida, 2015).

A recent investigation using MALDI-TOF fingerprinting found that *S. maltophilia* strain OK-5 harbored anti-FecI sigma factor (FecR) (Lee et al., 2017). On the other hand, a study identified a homolog of the ferric citrate receptor (FecA) in *S. maltophilia* strain WR-C (Huang and Lee Wong, 2007). Interestingly, the study found that unlike other Gram-negative bacteria such as *Escherichia coli* the *fecIR* regulatory genes are not located upstream of *fecA*. This suggest that the ferric citrate transport system in *S. maltophilia* may be regulated differently or the location of the regulators could be somewhere else. Our sequencing results revealed the “iron siderophore sensor protein (FeSS)” is corresponding to “iron dicitrate transport regulator FecR” (SMLT_RS18580) and “sigma factor ECF subfamily” is corresponding to “RNA polymerase sigma factor” (SMLT_RS12950) in strain K279a (Kalidasan et al., 2018a). Overall, iron regulation in *S. maltophilia* is potentially depended on Fur and sigma factors. However, it is essential to validate using expression profiles of regulatory knockout mutants or any other suitable approaches, to decipher on how these regulators directly control iron acquisition strategies in *S. maltophila*.

## Iron uptake and pathogenesis of *S. maltophilia*

Numerous studies have been reported on virulence properties, specifically investigating biofilm formation in *S. maltophilia* under normal nutritional status (Crossman and Dow, 2004; Huang et al., 2006; Passerini de Rossi et al., 2007; Pompilio et al., 2008, 2011; Biočanin et al., 2017; Liu et al., 2017; An and Tang, 2018). However, the correlation between iron and expression of virulence profiles among *S. maltophilia* has not been discussed extensively. Iron limitation was found to stimulate biofilm and extracellular polymeric substances (EPS) formation in *S. maltophilia*, resulting in less reactive oxygen species (ROS) production. Moreover, the study reported iron negatively regulates DSF production through Fur interaction and proved the expression of two iron-repressed OMPs (IROMPs), FepA, and TonB-dependent siderophore receptor. The killing assay using *Galleria mellonella* infection model showed spontaneous *fur* mutant was more virulent compared to wild-type (wt) strain *S. maltophilia* K279a. This contradicts with another study which revealed that iron repletion neither inhibits nor increases biofilm formation by *S. maltophilia* strain X26332 (Martinez et al., 2010). Such discrepancy in biofilm formation does not associate either with the phylogenetic connection or with the origin of isolates of *S. maltophilia* (Steinmann et al., 2018).

A study revealed that production of extracellular protease and chitinase by environmental *S. maltophilia* strain W81, were not altered even when the iron level was increased (Dunne et al., 1997). This showed the expression of extracellular enzyme among environmental strains are not affected by iron availability, due to the fact that soil contains a high amount of iron that are insoluble and not bioavailable (Berg et al., 1999). A similar trend can be observed in our study, whereby the environmental isolates did not show any significant differential expression for the iron acquisition targets when grown under both iron-depleted and iron-repleted conditions (Kalidasan et al., 2018a). It is important to note that, the amount of siderophore production and the strategies by which plants and microorganisms obtain iron from different sources, is likely to be highly variable under different environmental conditions or seasonally influenced by changes in carbon inputs into the rhizosphere during plant growth (Crowley, 2006). On the other hand, *S. maltophilia* was found to secrete hemolysin (Hly) (Garcia et al., 2002; Travassos et al., 2004; Thomas et al., 2014) which is important in the lysis of erythrocytes, thereby promoting the release of heme as iron sources for cellular growth (Runyen-Janecky, 2013). The hemolysin activity of Hly positive *S. maltophila* strains was inhibited with supplementation of ferric chloride (FeCl_3_) and the hemolytic activities were found similar to those of *Aeromonas caviae* and *Plesiomonas shigelloides* (Figueiredo et al., 2006). Furthermore, the study showed hemolysin production to be stimulated by Ca^2+^ ions but inhibited by EDTA, and in an overall modulated by iron. This finding suggests that synthesis of hemolysin is found to be iron regulated in most Gram-negative bacteria (Kim et al., 2009).

Under low iron level, it was found that *r*egulation of *p*athogenic *f* actors (*rpf*) cluster*, rpfF*, and *rpfB* in *S. maltophilia* strain WR-C are activated to synthesize DSFs, which stimulates iron uptake by FecA (Huang and Lee Wong, 2007). However, the study found that DSF has no effect on biofilm formation and synthesis of LPS, similarly reported in *Xanthomonas campestris* (Torres et al., 2007). Protease production and hemolytic activity in *S. maltophilia* were not modulated by DSF, but controlled by cyclic AMP (cAMP) receptor protein (CRP) (Kim et al., 2013). CRP responds to environmental changes, such as iron and glucose levels, and binds to the predicted CRP binding site upstream of *rpfF*, activating the *rpf* system. Moreover, *rpfF* was shown to affect siderophore production in *Xanthomonas oryzae* pv. *oryzae*, whereby the *rpfF* mutant strains were found unable to survive under low iron concentration (Chatterjee and Sonti, 2002). The FeoA family protein was found positively regulated by DSF in *S. maltophilia* R551-3, which plays important role in Feo system (Alavi et al., 2013). In short, the *rpf* and/or DSF system are involved in regulating various functional activities in *X. campestris* pv. campestris, including modulating iron uptake TonB-dependent proteins encoded by *tonB, bfeA, fepA, cirA, fyuA, iroN*, while *exbB, exbD1, exbD2, Xcc3216* are important for accessory proteins production (He et al., 2006).

## Conclusion and future directions

This review is important for understanding the mechanisms behind iron acquisition in *S. maltophilia*, it is, to our knowledge, the first of its kind to describe how *S. maltophilia* efficiently support its lifestyle as multi-drug-resistant global opportunistic nosocomial pathogen under iron availability. *S. maltophilia* potentially express three iron acquisition pathways which include, siderophore- and heme-mediated and Feo system under iron-limited condition. We regarded *S. maltophilia* as the “innocent culprit” as its represent potential benefits for biotechnological applications and simultaneously found to be associated with human and plant host. Iron was found to a crucial micronutrient for expression of various virulence profiles in *S. maltophilia*. Elaboration of these virulence factors may have clinical significance to the human host, especially among patient with immunocompromised conditions, increasing the difficulty in therapeutic approaches. In order to decipher complete iron acquisition systems in *S. maltophilia*, knockout mutants should be considered to understand the roles of differentially expressed targets during iron limitation. The effect of iron limitation on the proteome of *S. maltophilia* and mechanisms of Fur regulation are also interesting questions for future investigations.

## Author contributions

VK performs the literature search and wrote the manuscript. NJ proofreads the manuscript. SK, RA, and VN outline the idea and approve the final manuscript.

### Conflict of interest statement

The authors declare that the research was conducted in the absence of any commercial or financial relationships that could be construed as a potential conflict of interest.
